# Risk of pneumonia in the vicinity of goat farms: a comparative assessment of temporal variation based on longitudinal health data

**DOI:** 10.1186/s41479-023-00115-7

**Published:** 2023-09-05

**Authors:** C. Joris Yzermans, Youri P. Moleman, Peter Spreeuwenberg, Mark M.J. Nielen, Michel L.A. Dückers, Lidwien A.M. Smit, Christos Baliatsas

**Affiliations:** 1 Nivel (Netherlands Institute for Health Services Research), Utrecht, The Netherlands; 2 Institute for Risk Assessment Sciences (IRAS), Utrecht University, Utrecht, The Netherlands

**Keywords:** Pneumonia, Respiratory health, Temporal, Seasonal, Goat farming, Epidemiology

## Abstract

**Background:**

Although the association between living in the vicinity of a goat farm and the occurrence of pneumonia is well-documented, it is unclear whether the higher risk of pneumonia in livestock dense areas is season-specific or not. This study explored the temporal variation of the association between exposure to goat farms and the occurrence of pneumonia.

**Methods:**

A large population-based study was conducted in the Netherlands, based on electronic health records from 49 general practices, collected for a period of six consecutive years (2014–2019). Monthly incidence rates of pneumonia in a livestock dense area were compared with those of a control group (areas with low livestock density) both per individual year and cumulatively for the entire six-year period. Using individual estimates of livestock exposure, it was also examined whether incidence of pneumonia differed per month if someone lived within a certain radius from a goat farm, compared to residents who lived further away.

**Results:**

Pneumonia was consistently more common in the livestock dense area throughout the year, compared to the control area. Analyses on the association between the individual livestock exposure estimates and monthly pneumonia incidence for the whole six-year period, yielded a generally higher risk for pneumonia among people living within 500 m from a goat farm, compared to those living further away. Significant associations were observed for March (IRR 1.68, 95% CI 1.02–2.78), August (IRR 2.67, 95% CI 1.45–4.90) and September (IRR 2.52, 95% CI 1.47–4.32).

**Conclusions:**

The increased occurrence of pneumonia in the vicinity of goat farms is not season-specific. Instead, pneumonia is more common in livestock dense areas throughout the year, including summer months.

**Supplementary Information:**

The online version contains supplementary material available at 10.1186/s41479-023-00115-7.

## Background

Livestock farms are a potential source of chemical and microbial pollutants such as particulate matter, ammonia and endotoxins (cell wall components of Gram-negative bacteria) and have been linked to the spread of a variety of zoonotic (viral and bacterial) pathogens [1–3]. With a growing number of large-scale confined animal feeding operations, residents have been increasingly concerned about the effects on their health [4, 5].

A large body of epidemiological research has thoroughly studied the prevalence and incidence of (respiratory) health symptoms and conditions among residents living in the vicinity of livestock farms in the Netherlands, Germany and the United States, using electronic health records (EHR) of general practitioners (GPs), questionnaires, and pulmonary function tests [2, 4, 6–14]. Over the past decade, findings based on different methodological approaches showed that lower respiratory tract infections and acute respiratory symptoms such as coughing, shortness of breath and wheezing are significantly more common amongst residents living close to a livestock farm [5, 8, 10, 15–17], while in some cases a lower risk was observed for outcomes such as atopy and asthma [3, 8].

The most consistent finding however, independently of study design, time period, presence of other types of livestock and geographical region, has been the association between pneumonia and presence of goat farms primarily, and poultry farms to a lesser extent [2, 7, 10, 11, 13, 15, 18–20]. While the causal mechanisms behind this association remain unclear, there are important epidemiological aspects that have not been studied yet, such as the temporal variation of pneumonia in relation to livestock exposure. It is yet unknown whether the elevated risk is present year-round, or whether there is a monthly or seasonal variation in the risk of pneumonia in areas with a higher concentration of goat farms. The increased risk could also coincide with either peaks in pneumonia incidence during the winter season – typically from early December until the end of February in the Netherlands [21] – or with seasonal variation in goat farming practices such as lambing periods (mostly in February-May).

The main objective of this study is to fill this knowledge gap by examining the temporal variation in the association between exposure to goat farms and the occurrence of pneumonia as diagnosed by a general practitioner. Two methods were employed: An area comparison between exposed and control populations, and an analysis focusing on the exposed area by using individual exposure estimates.

## Methods

### Study design and population

This observational, population-based study assessed the incidence of pneumonia in a primary care-registered population in areas with and without a high livestock density. In the Netherlands every citizen is obliged to be registered on the list of just one general practice. The general practitioner acts as a gatekeeper to secondary care. Patient morbidity data are registered in Electronic Health Records (EHR) using the International classification of primary care (ICPC) case definitions [22]. For the present study, EHR were obtained for patients of 49 general practices, all located in municipalities of 30,000 inhabitants at most. Twenty-seven practices were located in the provinces of Limburg and Noord-Brabant, a goat farm dense area (exposed/livestock dense area) and 22 in control areas.

The “livestock dense area” comprises a highly populated rural area in the eastern part of the province of North Brabant and the northern part of the province of Limburg in the Netherlands. The high concentration of goat and other livestock farms and animals and the associated ambient endotoxin, particulate matter and ammonia levels in this area has been well-documented in recent studies [1, 3, 13, 23].

The “control area”, refers to a sample of various (rural) regions in the Netherlands with a similar degree of urbanization and low or no presence of livestock farms [3, 16].

### Outcome assessment

Incidence (number of newly diagnosed cases within a specific timeframe) of pneumonia (ICPC code: R81) was based on episodes of care [24], which include all patient encounters within a registered diagnostic (ICPC) code. These were obtained from pseudonymized electronic health records (EHR) in the Nivel Primary Care Database (Nivel-PCD). Dutch GPs diagnose pneumonia on the basis of clinical profile following a national guideline. In recent years they often use diagnostic tools such as oximeter and C-reactive protein. Laboratory determination is rarely requested.

### Estimates of individual exposure to goat farms in the livestock dense area

The presence of livestock farms was identified using “*Bestand Agrarische Bedrijfssituatie*” (BAB) - a national database, compiled by the Netherlands Enterprise Agency (RVO.nl). This database contains all the locations of companies known to RVO that are active in agriculture. Farm-level data from the year 2018 were available.

Registered patients’ home addresses in the livestock dense area were geo-coded: The locations of residence were derived from patient records, which were merged (under strict privacy regulations; see section on ethics approval) with coordinates on the location of companies in the BAB database to determine the distance from the home to the nearest farm. Only goat farms with at least 50 (dairy) goats were included in the analyses. This is the minimum number of animals required for a farm to be officially registered as such. Persons with incomplete addresses and/or health data were not included in the analysis. Following the approach of previous studies investigating health problems in the vicinity of goat farms [3, 7, 13], three binary variables were used corresponding to different “buffers”: Presence versus absence (reference category) of goat farms within a radius of 0-500 m, 0-1000 m and 0-2000 m from the home address.

### Statistical analysis

For the area comparison, the monthly incidence rates of pneumonia in the exposed area were compared with those of the control area, for a period of six consecutive years (2014–2019), both per individual month and cumulatively (all individual months combined) for the entire period. In addition, we analyzed pneumonia incidence for the whole six-year period (to increase power) among residents in the exposed area, using the individual estimates of livestock exposure.

Multilevel logistic and Poisson regression analyses were carried out, taking into account the hierarchical structure of the data (registered patients nested within general practices). The dependent variable in all analyses was the incidence of pneumonia, while the independent variable was the group type (livestock dense/exposed vs. control) for the area comparison and buffer (500 m, 1000 and 2000 m) for the analyses on individual livestock exposure estimates within the exposed area.

All analyses were adjusted for gender and age (polynomial, in order to allow for a potential nonlinear trend between age and morbidity) and registry duration (whether a person was registered at a general practice or not in that particular month - or year for the analyses over the six-year period). Analyses among residents of the exposed area were additionally adjusted for presence of poultry within 2000 m. Relative risks (RR) and incidence rate ratios (IRR) with 95% confidence intervals (CI) were generated, depending on the regression analysis type (pneumonia treated as dichotomous or count variable). A *p*-value < 0.05 was considered statistically significant. Analyses were performed with STATA versions 14.0 and 15.0 (StataCorp LP, College Station, TX, USA) and MLwiN (Centre for Multilevel Modelling, University of Bristol, Bristol, UK).

## Results

### Descriptive characteristics

**Fig. 1:**
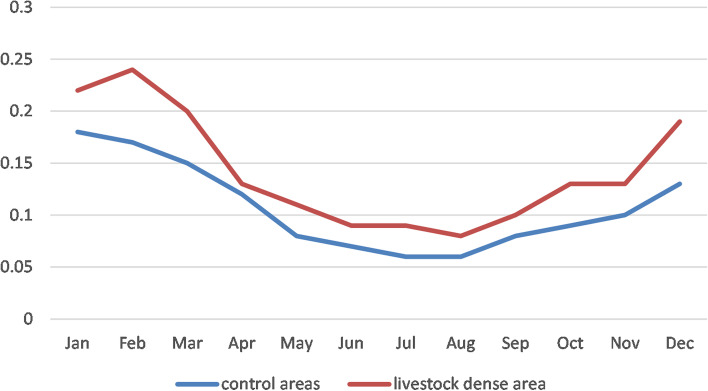
Average monthly incidence (%) of pneumonia in the livestock dense and control area for all years (2014–2019) combined

An overview of the sample size and participating general practices is presented in Table 1. In the livestock dense area, mean age of the total sample varied between 42.3 (SD 23.2) and 43.8 (SD 23.6) years, while the percentage of female patients ranged between 49.3% and 49.5% over the six year period. Similarly, the mean age of patients in the control area varied between 42.5 (SD 23.5) and 43.3 (SD 23.5) years and the percentage of female participants ranged between 49.4% and 49.9%. On a year basis, prevalence of pneumonia (proportion of people diagnosed at least once within each year) varied between 1.9% and 2.2% in the livestock dense area and between 1.3% and 1.6% in the control area, over the investigated period (Additional file 1: Figure A1). Most of the new pneumonia cases occurred between November and March in both comparison groups (Fig. 1).

About 30% of the sample in the livestock area (available for the analyses on individual exposure estimates) lived within 2000 m from a goat farm, 7% within 1000 m and approximately 1% within 500 m.

**Table 1:** Study population and number of general practices in the livestock dense and control area each year, included in the analyses on area comparison

Year	Total population in livestock dense area (N general practices)	Control population (N general practices)	Population in livestock dense area available for the analyses on individual exposure estimates (N general practices)
2014	112,985 (25)	75,389 (22)	96,784 (25)
2015	115,964 (26)	74,748 (22)	100,381 (26)
2016	116,089 (26)	76,919 (22)	105,330 (26)
2017	117,576 (27)	58,671 (19)	102,905 (26)
2018	109,947 (26)	47,090 (16)	93,223 (24)
2019	113,154 (25)	55,338 (18)	90,872 (23)

### Temporal variation of pneumonia risk in relation to goat farms

**Fig. 2:**
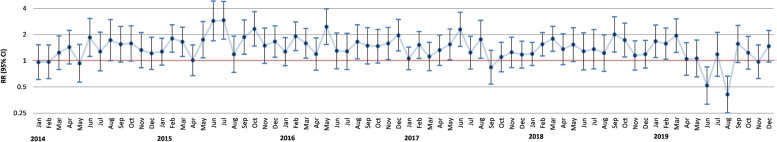
Differences in the monthly incidence of pneumonia between the livestock dense and control area for the period 2014–2019 (for additional details see Additional file 1: Table A1)

The risk of pneumonia was found to be consistently higher in the livestock dense area compared to the control group for most of the six year period (Fig. 2). Statistically significant differences in monthly incidence between the areas were often observed in February and March (for four of the investigated years) and also in June (for three years) (Fig. 2). The strongest relative risks were mainly observed in June (RRs ranging from 1.85 to 2.87) and also in July 2015 (RR 2.92) (Additional file 1: Table A1). Significant inverse associations (higher incidence in the control areas) were observed for two summer months in 2019 (June and August).

**Fig. 3:**
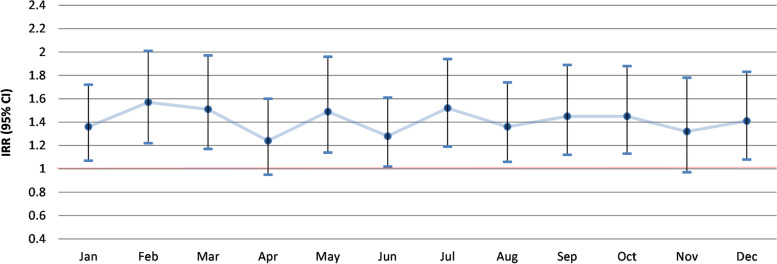
Differences between the livestock dense and control area in six year-combined monthly incidence of pneumonia (for additional details see Additional file 1: Table A2)

When we analyzed the incidence of pneumonia for every individual month combined for the whole 2014–2019 period, a significantly higher risk in the livestock dense area was found for ten months (Fig. 3), with incidence rate ratios ranging from 1.28 to 1.57 (Additional file 1: Table A2). Only for April and November differences in pneumonia rates were not statistically significant.

**Fig. 4:**
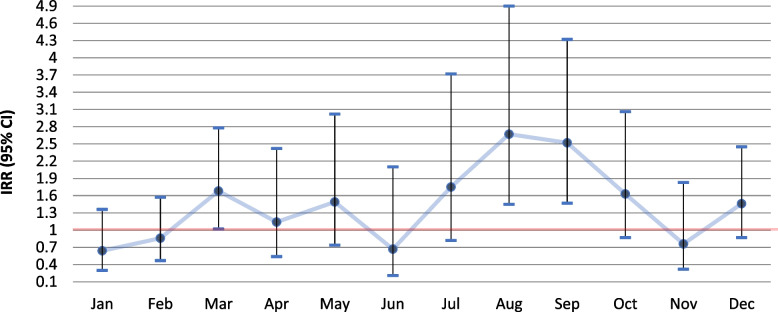
Differences in six year-combined monthly incidence of pneumonia for the period 2014–2019 between people living within 500 m from a goat farm vs. living further away in the livestock dense area (for additional details see Additional file 1: Table A3)

**Fig. 5:**
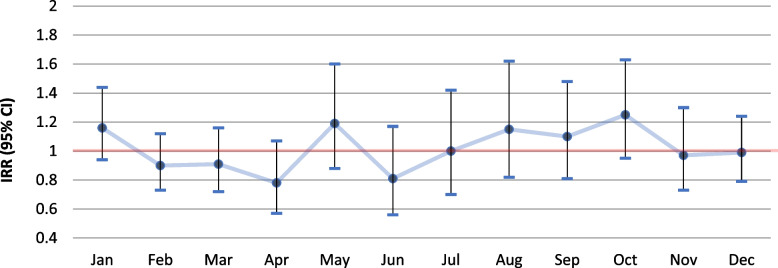
Differences in six year-combined monthly incidence of pneumonia for the period 2014–2019 between people living within 1000 m from a goat farm vs. living further away in the livestock dense area (for additional details see Additional file 1: Table A4)

**Fig. 6:**
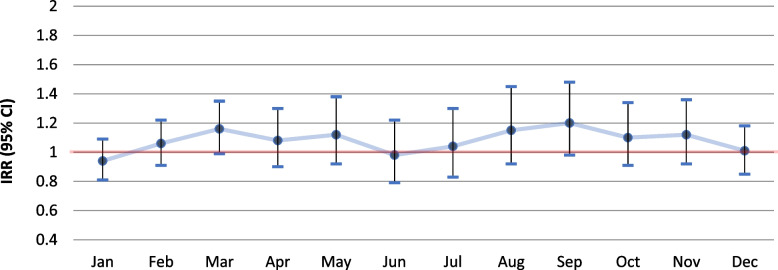
Differences in six year-combined monthly incidence of pneumonia (IRR) for the period 2014–2019 between people living within 2000 m from a goat farm vs. living further away in the livestock dense area (for additional details see Additional file 1: Table A5)

Analysis on the association between the individual livestock exposure estimates and monthly pneumonia incidence for the whole six-year period, showed a generally higher risk for pneumonia among residents of the livestock dense area living within 500 m from a goat farm (Fig. 4), for the largest part of the year, compared to those who did not. Significant associations were observed for March (IRR 1.68, 95% CI 1.02–2.78), August (IRR 2.67, 95% CI 1.45–4.90) and September (IRR 2.52, 95% CI 1.47–4.32) (Additional file 1: Table A3). No significant associations were observed for people living within 1000 or 2000 m from a goat farm (Figs. 5 and 6; Additional file 1: A4 & A5).

## Discussion

The present study focused on the temporal variation of pneumonia risk in an area with a high livestock farm density versus a control area. The risk of pneumonia was significantly higher throughout the year in the livestock dense area, in which the goat sector has been rapidly expanding over the years [25]. These findings were consistent over the investigated period of six consecutive years. When looking at the monthly incidence differences for every year apart, most of the significant area differences were observed for February and March. Nevertheless, after combining all years together, area differences were significant for most of the months. When only focusing on individual exposure estimates in the livestock dense area it was found that the risk for pneumonia was highest and most often significant among people living within 500 m from a goat farm, compared to those who did not. On the basis of the current findings there is no clear indication that the higher pneumonia rates in the livestock dense area are month- or season-specific and/or coincide with the lambing season at goat farms or increased incidence of respiratory viral infections during the winter months. In addition, the pattern of seasonal distribution of pneumonia for the combined six-year period was similar in the two comparison groups, with many new cases occurring during the winter months.

To our knowledge, this is the first study that explored temporal variation of pneumonia in relation to livestock exposure. The mechanism underlying the observed associations between pneumonia and the presence of livestock—and more specifically goat farms—in the living environment remains unclear. However, our study suggests that risk factors or etiological agents are present throughout the year, and observed associations are not restricted to a specific season. A potential pathway could be related to susceptibility to respiratory infections after being exposed to farm emissions such as fine dust, mold, endotoxins and ammonia [26, 27]. Prior infection with *Coxiella burnetii* – a zoonotic bacterial pathogen responsible for outbreaks of respiratory infections near goat farms in the Netherlands between 2007 and 2010 [28]– could also contribute to prolonged susceptibility to respiratory problems [29–31], although population-based studies have not shown significant associations between positive serological tests for *Coxiella burnetii* and history of pneumonia [10, 20]. Future research on the microbiological profile of pneumonia patients in livestock dense areas and unexposed control areas could provide new insights in the pathogenic agents responsible for the consistently higher pneumonia risk [32]. Moreover, epidemiological assessment of exposure–response associations between combinations of various ambient environmental pollutants would enable the identification of possible joint exposure effects and contributing components on pneumonia risk. In the long run, exploration of exposomal [33–35] influences on relevant health outcomes would lead to a better recognition and comprehension of the impact of individual and environmental factors on respiratory health, in the context of livestock farming.

Among the strengths of the current study are the use of objective health data based on EHR in a large patient population over multiple consecutive years, as well as the use of two approaches to assess risk of pneumonia in relation to the living environment: (i) an area comparison and (ii) a closer focus on the livestock dense area using objective estimates of livestock exposure. Among the limitations is that livestock farm locations in 2018 were used as a proxy of farm-related exposures for the whole six year period (2014–2019). In addition, although livestock farms in the study area can be important sources of microbial agents such as endotoxins [1], the association of pneumonia with emissions of specific pollutants and pathogens was not assessed in this study. Misclassification of pneumonia diagnosis could also be a possibility for some cases. Furthermore, information on several possible confounders was not available, such as exposure to other sources of air pollution (from traffic and industry), smoking habits and socioeconomic status (SES). In earlier studies in the same livestock dense area, correction for SES indicators and smoking did not alter the observed associations [10, 36]. Finally, data on occupational exposure was not available. However, on the basis of previous studies in the same area, this concerns a small fraction of the study population and exclusion of residents living or working on a livestock farm did not alter the findings [3, 8, 11].

## Conclusions

This large population-based study shows that pneumonia is more common in a livestock dense area than in a rural control area, and this association was present throughout the year. A year-round increased risk was also observed in residents of the livestock dense area living within a 500 m radius of a goat farm, compared to those living further away. This study provides evidence that associations between livestock farm exposures and pneumonia are not season-specific, suggesting that potential risk factors or etiological agents should be present over the course of the whole year.

### Supplementary Information

**Additional file 1: Figure A1.** Annual prevalence (%) of pneumonia in the livestock dense and control area over the investigated period. **Table A1.** Detailed estimates (RR, 95% CI) of the differences in the monthly incidence of pneumonia between the livestock dense and control area for the period 2014-2019 (significant differences in bold). **Table A2.** Detailed estimates (IRR, 95% CI) of the differences between the livestock dense and control area in six year-combined monthly incidence of pneumonia (significant differences in bold). **Table A3.** Detailed estimates (IRR, 95% CI) of the association between presence (yes/no) of goat farms within a range of 500 m in the livestock dense area and six year-combined monthly incidence of pneumonia (IRR) for the period 2014-2019 (significant differences in bold). **Table A4.** Detailed estimates (IRR, 95% CI) of the association between presence (yes/no) of goat farms within a range of 1000 m in the livestock dense area and six year-combined monthly incidence of pneumonia (IRR) for the period 2014-2019. **Table A5.** Detailed estimates (IRR, 95% CI) of the association between presence (yes/no) of goat farms within a range of 2000 m in the livestock dense area and six year-combined monthly incidence of pneumonia (IRR) for the period 2014-2019

## Data Availability

In consultation with the Medical Ethical Committee that approved the study protocol, data from the VGO study are not publicly available due to privacy protection of participants. The study’s privacy regulations state that only researchers from Nivel, IRAS, and RIVM (consortium partners) have access to the study database. Sharing an anonymized and de-identified dataset is not possible as it would still contain Electronic Health Records and the personal data of participants, which could potentially lead to the identification of subjects. Researchers may contact the data access committee through Remco Coppen, PhD, LLM (r.coppen@nivel.nl) or non-personal departmental email (NIVEL: zorgregistraties@nivel.nl) or contact Christos Baliatsas, PhD (c.baliatsas@nivel.nl), Joris Yzermans, PhD (J.Ijzermans@nivel.nl), or Michel Duckers, PhD (m.duckers@nivel.nl).

## Abbreviations

CI: Confidence interval
EHR: Electronic health records
GPs: General practitioners
IRR: Incidence rate ratio
PCD: Primary care database
RR: Relative risk
