# Supplementary material for: Risk of pneumonia in the vicinity of goat farms: a comparative assessment of temporal variation based on longitudinal health data
Source: Pneumonia (Nathan). 2023 Sep 5;15:13. doi: 10.1186/s41479-023-00115-7 (PMC10478456; doi:10.1186/s41479-023-00115-7)
Supplement: Supplementary file 1 — Additional file 1: Figure A1. Annual prevalence (%) of pneumonia in the livestock dense and control area over the investigated period. Table A1. Detailed estimates (RR, 95% CI) of the differences in the monthly incidence of pneumonia between the livestock dense and control area for the period 2014-2019 (significant differences in bold). Table A2. Detailed estimates (IRR, 95% CI) of the differences between the livestock dense and control area in six year-combined monthly incidence of pneumonia (significant differences in bold). Table A3. Detailed estimates (IRR, 95% CI) of the association between presence (yes/no) of goat farms within a range of 500 m in the livestock dense area and six year-combined monthly incidence of pneumonia (IRR) for the period 2014-2019 (significant differences in bold). Table A4. Detailed estimates (IRR, 95% CI) of the association between presence (yes/no) of goat farms within a range of 1000 m in the livestock dense area and six year-combined monthly incidence of pneumonia (IRR) for the period 2014-2019. Table A5. Detailed estimates (IRR, 95% CI) of the association between presence (yes/no) of goat farms within a range of 2000 m in the livestock dense area and six year-combined monthly incidence of pneumonia (IRR) for the period 2014-2019 [file 41479_2023_115_MOESM1_ESM.docx]

**Supplementary Information**

**Appendix**

**Figure A1** Annual prevalence (%) of pneumonia in the livestock dense and control area over the investigated period


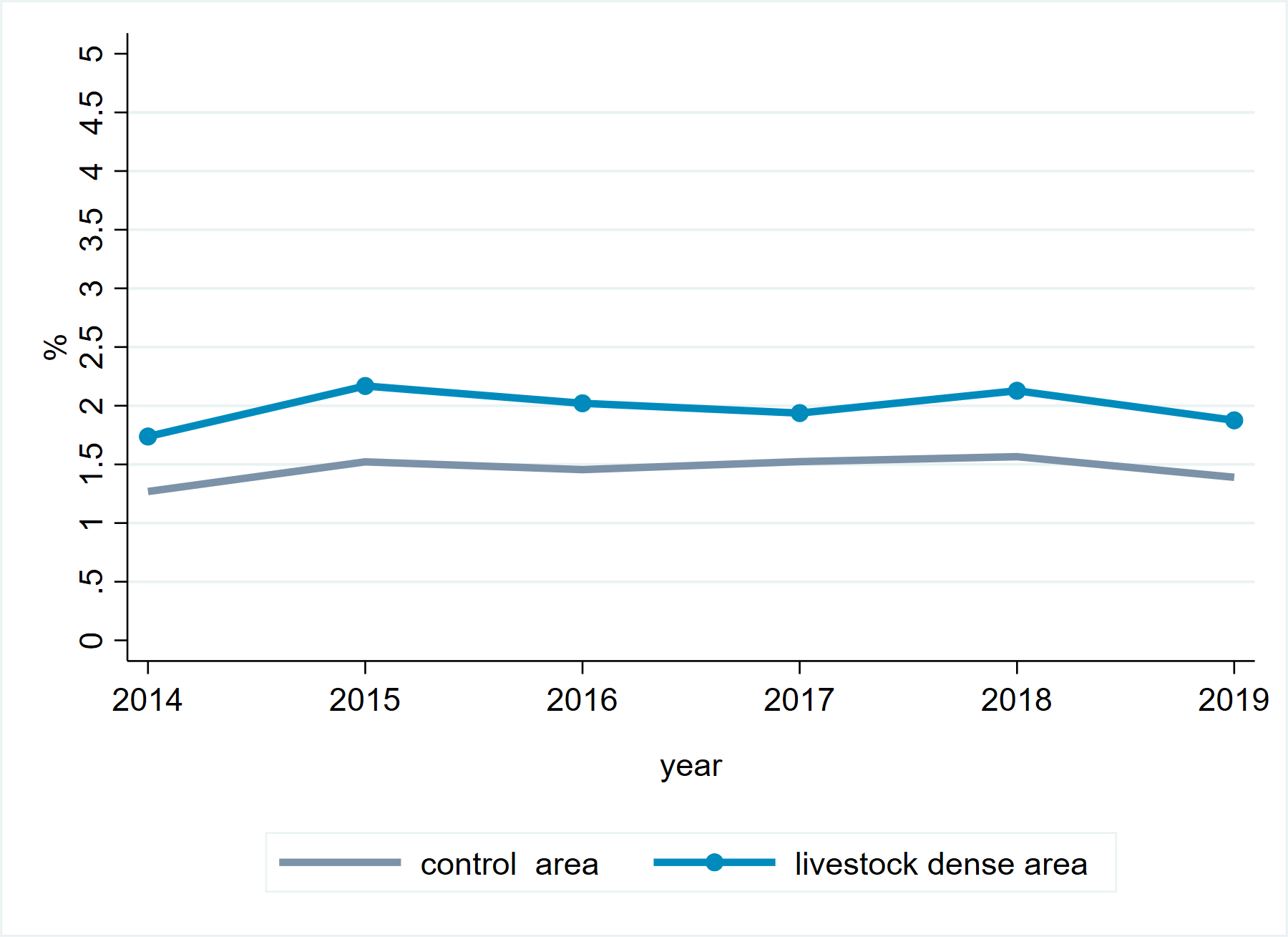


**Table A1** Detailed estimates (RR, 95% CI) of the differences in the monthly incidence of pneumonia between the livestock dense and control area for the period 2014-2019 (significant differences in bold)

|  | 2014 | 2015 | 2016 | 2017 | 2018 | 2019 |
| --- | --- | --- | --- | --- | --- | --- |
| January | 0.96 (0.61-1.53) | 1.27 (0.89-1.82) | 1.27 (0.88-1.84) | 1.06 (0.79-1.43) | 1.20 (0.88-1.62) | **1.67 (1.09-2.58)** |
| February | 0.97 (0.62-1.51) | **1.80 (1.25-2.59)** | **1.91 (1.31-2.80)** | **1.52 (1.06-2.17)** | **1.55 (1.14-2.11)** | **1.57** (**1.04-2.38)** |
| March | 1.24 (0.79-1.93) | **1.65 (1.12-2.43)** | **1.58 (1.07-2.35)** | 1.12 (0.77-1.63) | **1.79** **(1.28-2.49)** | **1.94** **(1.24-3.03)** |
| April | 1.43 (0.92-2.23) | 1.01 (0.67-1.52) | 1.19 (0.78-1.83) | 1.32 (0.89-1.97) | 1.36 (0.90-2.04) | 1.05 (0.86-1.61) |
| May | 0.93 (0.57-1.54) | **1.75 (1.08-2.83)** | **2.46 (1.53-3.95)** | **1.54 (1.02-2.32)** | 1.53 (0.98-2.40) | 1.06 (0.65-1.73) |
| June | **1.85 (1.13-3.06)** | **2.87 (1.70-4.86)** | 1.30 (0.81-2.08) | **2.29** **(1.46-3.60)** | 1.28 (0.78-2.09) | **0.52 (0.31-0.85)** |
| July | 1.28 (0.76-2.13) | **2.92 (1.77-4.81)** | 1.28 (0.79-2.07) | 1.24 (0.80-1.91) | 1.36 (0.81-2.28) | 1.18 (0.66-2.12) |
| August | **1.72 (1.00-2.98)** | 1.19 (0.73-1.92) | **1.65 (1.05-2.60**) | **1.76 (1.07-2.91)** | 1.22 (0.75-1.99) | **0.41** **(0.25-0.66)** |
| September | 1.55 (0.96-2.48) | **1.87 (1.19-2.95)** | 1.49 (0.92-2.40) | 0.84 (0.54-1.32) | **2.01 (1.26-3.20)** | 1.56 (0.95-2.55) |
| October | 1.58 (0.99-2.52) | **2.32 (1.47-3.67)** | 1.47 (0.94-2.29) | 1.10 (0.75-1.63) | **1.73 (1.10-2.71)** | 1.24 (0.80-1.91) |
| November | 1.32 (0.83-2.11) | 1.49 (0.93-2.38) | **1.58 (1.03-2.42)** | 1.25 (0.83-1.87) | 1.15 (0.78-1.69) | 0.97 (0.62-1.52) |
| December | 1.22 (0.79-1.86) | **1.66 (1.09-2.52)** | **1.96 (1.29-2.98)** | 1.18 (0.82-1.68) | 1.19 (0.82-1.71) | 1.47 (0.97-2.22) |

**Table A2** Detailed estimates (IRR, 95% CI) of the differences between the livestock dense and control area in six year-combined monthly incidence of pneumonia (significant differences in bold)

|  | Total period 2014-2019 |
| --- | --- |
| January | **1.36 (1.07-1.72)** |
| February | **1.57 (1.22-2.01)** |
| March | **1.51 (1.17-1.97)** |
| April | 1.24 (0.95-1.60) |
| May | **1.49 (1.14-1.96)** |
| June | **1.28 (1.02-1.61)** |
| July | **1.52 (1.19-1.94)** |
| August | **1.36 (1.06-1.74)** |
| September | **1.45 (1.12-1.89)** |
| October | **1.45 (1.13-1.88)** |
| November | 1.32 (0.97-1.78) |
| December | **1.41 (1.08-1.83)** |

**Table A3** Detailed estimates (IRR, 95% CI) of the association between presence (yes/no) of goat farms within a range of 500 m in the livestock dense area and six year-combined monthly incidence of pneumonia (IRR) for the period 2014-2019 (significant differences in bold)

|  | Total period 2014-2019 |
| --- | --- |
| January | 0.64 (0.30-1.36) |
| February | 0.86 (0.47-1.57) |
| March | **1.68 (1.02-2.78)** |
| April | 1.14 (0.54-2.42) |
| May | 1.49 (0.74-3.02) |
| June | 0.67 (0.21-2.10) |
| July | 1.75 (0.82-3.72) |
| August | **2.67 (1.45-4.90)** |
| September | **2.52 (1.47-4.32)** |
| October | 1.63 (0.87-3.06) |
| November | 0.76 (0.32-1.83) |
| December | 1.46 (0.87-2.45) |

**Table A4** Detailed estimates (IRR, 95% CI) of the association between presence (yes/no) of goat farms within a range of 1000 m in the livestock dense area and six year-combined monthly incidence of pneumonia (IRR) for the period 2014-2019

|  | Total period 2014-2019 |
| --- | --- |
| January | 1.16 (0.94-1.44) |
| February | 0.90 (0.73-1.12) |
| March | 0.91 (0.72-1.16) |
| April | 0.78 (0.57-1.07) |
| May | 1.19 (0.88-1.60) |
| June | 0.81 (0.56-1.17) |
| July | 1.00 (0.70-1.42) |
| August | 1.15 (0.82-1.62) |
| September | 1.10 (0.81-1.48) |
| October | 1.25 (0.95-1.63) |
| November | 0.97 (0.73-1.30) |
| December | 0.99 (0.79-1.24) |

**Table A5** Detailed estimates (IRR, 95% CI) of the association between presence (yes/no) of goat farms within a range of 2000 m in the livestock dense area and six year-combined monthly incidence of pneumonia (IRR) for the period 2014-2019

|  | Total period 2014-2019 |
| --- | --- |
| January | 0.94 (0.81-1.09) |
| February | 1.06 (0.91-1.22) |
| March | 1.16 (0.99-1.35) |
| April | 1.08 (0.90-1.30) |
| May | 1.12 (0.92-1.38) |
| June | 0.98 (0.79-1.22) |
| July | 1.04 (0.83-1.30) |
| August | 1.15 (0.92-1.45) |
| September | 1.20 (0.98-1.48) |
| October | 1.10 (0.91-1.34) |
| November | 1.12 (0.92-1.36) |
| December | 1.01 (0.85-1.18) |
